# Human self and Neurosurgery: Advances and insights from Geneva

**DOI:** 10.1016/j.bas.2025.104385

**Published:** 2025-08-06

**Authors:** Abdullah Al Awadhi, Daniel Kiss-Bodolay, Simone Grannò, Roberta Ronchi, Eva Bobbink-Blondiaux, Rémi Tyrand, Colette Boëx, Philippe Voruz, Giannina Rita Iannotti, Julie Péron, Bruno Herbelin, Olaf Blanke, Karl Schaller

**Affiliations:** a Division of Neurosurgery, Department of Clinical Neuroscience, University Hospitals of Geneva (HUG), Switzerland; b General and Cognitive Neurology Unit, Division of Neurology, Department of Clinical Neuroscience, University Hospitals of Geneva (HUG), Switzerland; c NeuroCentre, University Hospitals of Geneva (HUG), Switzerland; d Clinical and Experimental Neuropsychology Laboratory, Department of Psychology, University of Geneva, Switzerland; e Laboratory of Cognitive Neuroscience, Neuro-X Institute, École Polytechnique Fédérale de Lausanne (EPFL), Campus Biotech, Geneva, Switzerland

**Keywords:** Self-consciousness, Neurosurgery, Onco-functional balance, Interoception, Self-other voice discrimination (SOVD), Heartbeat-evoked potentials (HEPs)

## Abstract

**Introduction:**

The preservation of the human self—a fundamental yet underexplored aspect of neurosurgical practice—has gained increasing attention in recent years.

**Research question:**

How can neural correlates of self-consciousness be identified, monitored, and protected during brain tumor surgery, and how might this reshape the concept of “onco-functional balance”?

**Material and methods:**

This review synthesizes emerging evidence from neuroimaging, neuropsychology, and intraoperative neurophysiology to build a framework for integrating the concept of self into modern neurosurgical practice.

**Results:**

We describe the anatomical and functional basis of bodily and cognitive self-awareness, highlighting the roles of interoception, multisensory integration, and higher-order cortical networks such as the medial prefrontal cortex, insula and temporoparietal junction. We outline perioperative tools for clinical assessment, including validated scales for anosognosia and disownership, as well as the Self-Other Voice Discrimination (SOVD) paradigm and Heartbeat-Evoked Potentials (HEPs), which offer quantifiable markers of self-processing.

**Discussion and conclusion:**

We argue for a reconceptualization of “eloquent” cortex to include regions critical for the preservation of self. As neurosurgery advances toward precision-guided, patient-centered care, protecting the self must become an explicit goal alongside motor, sensory, and language preservation. Future directions include real-time intraoperative monitoring of HEPs, development of functional risk maps for self-related structures, and broader implementation of personalized, neurocognitive surgical planning.

Ultimately, this work proposes a shift from an “onco-functional” to an “onco-functional-identity” paradigm—where the integrity of the patient's personality, agency, and awareness becomes a measurable, preservable endpoint of neurosurgical care.

## Highlights

• Mapping the neural basis of self is becoming feasible in neurosurgical procedures.
• Onco-functional balance may evolve into an onco-functional-identity paradigm.
• Self-Other Voice Discrimination identifies self-related deficits in neurosurgical patients.
• Heartbeat-Evoked Potentials offer biomarkers for interoception and self-consciousness.
• Functional imaging highlights the medial prefrontal cortex as central to self-awareness.

## Abbreviations

ACC: anterior cingulate cortex
CMS: cortical midline structures
DMN: default mode network
DTI: diffusion tensor imaging
EBA: extrastriate body area
ECG: electrocardiogram
EEG: electroencephalography
fMRI: functional magnetic resonance imaging
HER: Heartbeat-Evoked Response
HEP: Heartbeat-Evoked Potential
iMRI: intraoperative magnetic resonance imaging
IPS: intraparietal sulcus
IONM: intraoperative neuromonitoring
MEG: magnetoencephalography
MEPs: motor evoked potentials
MST: medial superior temporal area
mPFC: medial prefrontal cortex
PCC: posterior cingulate cortex
PIVC: parieto-insular vestibular cortex
PMC: premotor cortex
SCEPs: subcortico-cortical evoked potentials
SEEG: stereo-electroencephalography
SMA: supplementary motor area
SOVD: Self-Other Voice Discrimination
SSEPs: somatosensory evoked potentials
TPJ: temporoparietal junction
VIP: ventral intraparietal area
vmPFC: ventromedial prefrontal cortex

## 1 Introduction

“Who am I? How am I feeling?” The emergence of human self is one of the most complex topics of neuroscience. It comprises a vast array of neuropsychological concepts related to one's being, ranging from feelings to personality. As far back as 1890, William James described the self by subdividing it into different domains, such as the *Material Self* which includes one's body, and the *Spiritual Self*, which encompasses a person's inner faculties, including moral sensibility, cognition and thought processes, which are integral of one's personality (James, 1890). The conscious and subconscious aspects of self have been studied extensively for centuries across different fields of neuroscience, including but not limited to basic neuroscience, neuropsychology, neuroimaging and neurophysiology.

Self-consciousness holds an important role in neurosurgery, where patients with self-altering pathologies are encountered every day. In the context of brain surgery and especially for brain tumor removal, as is avoiding any postoperative neurological deficits, maintaining a patient's sense of self is as crucial as the primary surgical goal. In brain tumor surgery, the concept of “onco-functional balance” emerges as a critical objective. This delicate balance entails maximizing the resection of tumor tissue while meticulously preserving critical brain structures that are intimately linked to the patient's neurological function. The following article will focus on the preservation of the sense of self, a set of neurological functions that has been long overlooked in the field of neurosurgery. Indeed, there exist many reports of neurosurgical outcomes with change of the patient's personality after the procedure (Orepic et al., 2023). This preservation is essential not just for physical health but for maintaining the continuity of the patient's identity, personality, and cognitive function.

Advancements in neuroimaging have significantly enhanced our ability to map and visualize brain regions, allowing surgeons to delineate tumor boundaries with greater precision. Techniques such as functional magnetic resonance imaging (fMRI) and diffusion tensor imaging (DTI) provide detailed insights into the brain's functional architecture and white matter tracts, enabling the identification of areas critical to the self (Northoff et al., 2006). These may also be used intraoperatively via augmented and mixed reality technologies injected into the surgical microscope to assess the spatial relationship between brain eloquent structures and the lesion (Haemmerli et al., 2021).

In parallel, neurophysiological monitoring techniques, like intraoperative brain mapping and neuromonitoring through surface, cortical and subcortical evoked potentials, allow real-time assessment of functional areas during surgery, helping to avoid damage to critical brain regions. On the one hand, mapping and neuromonitoring are routinely used under general anesthesia for motor tracts (Krieg et al., 2013; Raabe et al., 2014), as well as for language (Kabir et al., 2023; Mariani et al., 2021; Yamao et al., 2014) and visual pathways (Boëx et al., 2021a). On the other hand, aspects related to personality and the sense of self are not routinely monitored in patients under general anesthesia, despite their significant impact on quality of life (Bonosi et al., 2023; Schaller et al., 2021).

The extent of surgical resection can lead to various postoperative deficits: insular lesions may cause anxiety, hyperventilation, and emotional instability (de Morree et al., 2016; Ronchi et al., 2015); frontal lesions are associated with alexithymia and personality changes (Campanella et al., 2014); and temporal-parietal lesions can result in impairments in theory of mind—the ability to understand one's own and others' mental states—as well as reduced self-awareness (Campanella et al., 2014). Given the potential for these consequences to severely affect both patients and their families, it is crucial for neurosurgeons to implement tools and strategies aimed at safeguarding regions responsible for essential cognitive and emotional functions and the neural substrates of self-awareness and personality.

In Geneva, recent innovations in these fields demonstrate a commitment to refining surgical approaches that respect the patient's self while enhancing oncological outcomes. The following article will delve into these technological and methodological advancements, showcasing how they contribute to achieving the “onco-functional balance” of self and ensuring that patients retain their sense of identity and autonomy post-surgery.

## 2 Background

### 2.1 Definition of self

To date, several theoretical models have been proposed in the context of the self. Nevertheless, what they have in common is the fact that the human sense of self is a multifaceted phenomenon. One of the main hypotheses is that the self arises from the interaction of both cognitive processes—such as self-awareness, self-concept, and reflective thought—and bodily mechanisms, particularly through autonomic interoception, or the perception of internal physiological states.

Building on William James’ early distinction between the “I” and the “Me,” the self can be conceptualized as comprising two interrelated domains: the cognitive self and bodily self-consciousness (James, 1890). The former involves abstract self-representation, autobiographical memory, and metacognition, while the latter refers to the immediate, embodied sense of being a self in space. Bodily self-consciousness arises through the integration of multisensory inputs, including exteroceptive (visual, tactile, proprioceptive) and interoceptive signals. This integration forms a pre-reflective bodily representation of the self in the brain (Northoff et al., 2006; Blanke et al., 2015).

The somatic marker hypothesis, introduced by Antonio Damasio, further links interoception and self-awareness by proposing that emotional feelings are shaped by representations of bodily states in response to salient stimuli (Damasio et al., 2000). These “somatic markers” are generated in regions such as the anterior insula and orbitofrontal cortex, which not only support interoceptive awareness but also contribute to personality traits, decision-making, and social behavior (Northoff et al., 2006; Blanke et al., 2015; Craig, 2002; Critchley et al., 2004; Seeley et al., 2007). As such, interoceptive and emotional self-related processes are tightly interwoven, with shared neural substrates and behavioral outcomes.

Interoception is a form of bodily awareness that encompasses both conscious and unconscious monitoring of internal functions, such as cardiac, respiratory, and digestive signals (Craig, 2002). The central autonomic network (CAN) supports this integration, linking the brain to peripheral organ systems via afferent and efferent pathways. The CAN includes brainstem and hypothalamic structures, as well as limbic and paralimbic areas such as the insula, anterior cingulate cortex (ACC), amygdala, and hippocampus—regions also critical for affective and motivational regulation (Orepic et al., 2023). These structures receive afferent input from visceral organs and modulate bodily states, forming a crucial interface between homeostatic regulation and the construction of self-experience (Craig, 2002; Critchley et al., 2004).

Taken together, these frameworks suggest that the self is not an abstract, disembodied construct, but rather a sensorimotor and affective entity grounded in the body. Within this embodied self, three key dimensions of bodily self-consciousness have been delineated: self-identification (the experience of owning a body), self-location (the perceived spatial position of the self), and the first-person perspective (the subjective origin of perception in space)(Blanke, 2012).

These components are supported by distinct but overlapping multisensory brain networks, and they form the foundation for the sections that follow.

### 2.2 Self-identification

The experience of body ownership—the sense that one's body or body parts belong to oneself—is a foundational component of bodily self-consciousness. A widely used experimental paradigm to study this is the rubber hand illusion (RHI), in which participants perceive a fake hand as their own when synchronous stroking is applied to both the real and fake hand (Botvinick and Cohen, 1998; Ehrsson et al., 2004). This illusion has been shown to activate a distributed multisensory network involving the premotor cortex (PMC), intraparietal sulcus (IPS), sensorimotor cortex, and insula (Ehrsson et al., 2005; Tsakiris et al., 2007; Kanayama et al., 2007). Other regions, such as the supplementary motor area (SMA), posterior parietal cortex, ACC, and cerebellum, also show increased activity during the illusion (Lloyd et al., 2006; Ehrsson et al., 2007).

These brain areas form a multisensory integration network, particularly the PMC and IPS, where neurons respond to combinations of visual, tactile, and proprioceptive inputs (Graziano and Gandhi, 2000; Graziano et al., 1997; Iriki et al., 1996). This trimodal convergence allows for flexible updating of body ownership representations based on incoming sensory information, which is essential for self-identification.

To extend these findings beyond limb-specific ownership, researchers have adapted these paradigms to whole-body illusions using head-mounted displays (HMDs). In these experiments, participants view a virtual or filmed avatar of themselves from a third-person perspective while tactile stimuli are applied synchronously to both their physical body and the virtual body (Lenggenhager et al., 2007; Petkova and Ehrsson, 2008). Such setups reliably induce a sensation of full-body self-identification with the avatar.

These full-body illusions engage overlapping but distinct neural circuits compared to the RHI, again involving the PMC and IPS, but also recruiting the extrastriate body area (EBA), sensorimotor cortex, and temporo-parietal cortex (Blanke et al., 2015; Blanke, 2012). These findings emphasize that self-identification depends on the coherent integration of multiple sensory modalities within a specialized neural architecture that extends beyond localized body parts to the representation of the body as a whole (Aspell et al., 2009).

### 2.3 Self-location and the first-person perspective

Self-location—the experience of where one feels located in space—is tightly coupled with the first-person perspective, the spatial origin from which one perceives the world. While closely related to self-identification, these components rely on partially distinct multisensory mechanisms and neural circuits.

Experimental manipulations using HMDs have demonstrated that the perceived location of the self can be experimentally shifted away from the physical body. In setups where participants lie supine and view their body from above while receiving synchronous tactile stimulation, individuals often report an illusory displacement of their self-location towards the position of the seen body (Ehrsson et al., 2007; Lenggenhager et al., 2007).

Whereas self-identification depends on visuo-tactile and proprioceptive integration, self-location and first-person perspective involve the integration of visuo-tactile and vestibular signals. These are mediated by trimodal neurons found in multisensory areas such as the ventral intraparietal area (VIP), parieto-insular vestibular cortex (PIVC), and middle superior temporal area (MST)—regions involved in spatial orientation and body position in extrapersonal space (Graziano et al., 1997; Bremmer et al., 2002).

In addition, illusory self-location has been associated with activation of the posterior superior temporal gyrus (pSTG), medial premotor cortex (mPMC), somatosensory cortex, and medial prefrontal cortex (mPFC)(Ionta et al., 2011). Notably, lesions in the temporoparietal junction (TPJ) can lead to disembodied experiences and out-of-body phenomena, highlighting its key role in integrating multisensory signals for spatially localized self-consciousness (Blanke et al., 2002; Ridder et al., 2007; Blondiaux et al., 2021).

Together, these findings suggest that self-location and the first-person perspective are constructed through a dynamic process of multisensory integration, wherein vestibular, somatosensory, and visual inputs converge to determine where and from what vantage point we experience ourselves to exist.

### 2.4 Self-agency

Another fundamental aspect of self-consciousness is self-agency—the subjective experience of being the initiator of one's own actions, thoughts, or movements. This sense of agency is closely linked to predictive motor control: the brain compares internally generated predictions about sensory outcomes (e.g., from movement or speech) with actual feedback, producing the feeling of volitional authorship when they match (Blanke et al., 2015; Seth, 2013). Neural substrates consistently associated with self-agency include the pre-SMA, mPFC, insula, and TPJ (Blanke et al., 2015, Park and Blanke, 2019).

### 2.5 Interoception

Interoception—the sense of the internal physiological state of the body—relies on a distributed network of brain regions that integrate bodily signals with emotional and cognitive processes. Across multiple neuroimaging studies, the right anterior insula consistently emerges as a central hub for interoceptive awareness and accuracy, particularly in tasks involving heartbeat perception (Critchley et al., 2004; Garfinkel and Critchley, 2013). This region plays a key role in integrating bodily sensations with subjective emotional experience (Zaki et al., 2012).

The ACC is frequently co-activated with the insula and is thought to support attentional modulation and cognitive integration of interoceptive information (Khalsa et al., 2009; Pollatos et al., 2007). The ventromedial prefrontal cortex (vmPFC) also contributes to these processes, especially in the integration of emotional and bodily awareness (Terasawa et al., 2013).

A dual-pathway model has been proposed in which interoceptive signals are processed both via somatosensory afferents from the body and through a higher-order insula/ACC-centered network (Khalsa et al., 2009). Supporting this, distinct neural substrates have been identified for visceral versus somatosensory interoception: the left ACC is associated with visceral signals, while somatosensory interoception correlates with white matter integrity in several tracts, including the anterior thalamic radiation and corticospinal tract (Boccia et al., 2023).

Oscillatory activity and functional connectivity analyses further support a functional differentiation between interoceptive and exteroceptive attention, with interoception engaging higher-frequency oscillations in regions such as the insula, amygdala, and somatosensory cortices (García-Cordero et al., 2017). These findings suggest that interoception is not only multimodal but also involves both conscious and implicit processes, as evidenced by the identification of an intrinsic allostatic-interoceptive system spanning the dorsal insula and visceromotor regions (Kleckner et al., 2017).

Recent work has shown that interoceptive signals, such as heartbeat cues, can directly influence full-body illusions by modulating self-identification and self-location. In two studies using synchronized cardio-visual stimulation, participants experienced stronger embodiment toward a virtual body, supporting a link between internal physiological states and altered bodily self-consciousness (Aspell et al., 2013; Heydrich et al., 2018). Together, this body of evidence underscores the complexity of interoceptive processing and its foundational role in bodily self-awareness, emotional experience, and higher-order self-representation.

**Fig. 1:**
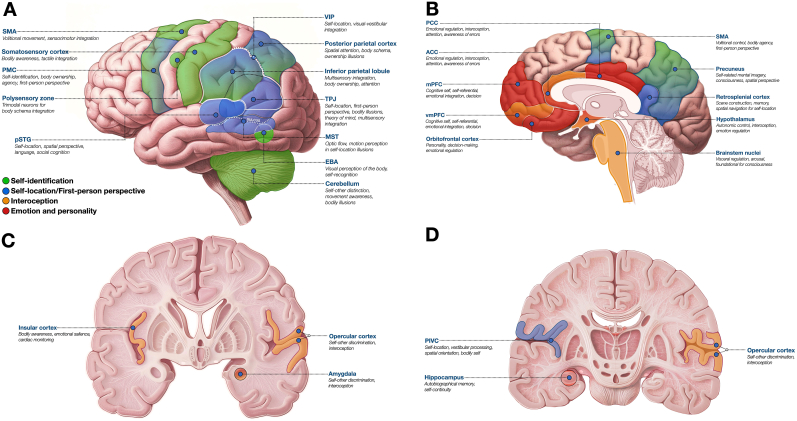
Representations of the brain showing the main cortical areas and other structures holding functions related to the self. **A** Lateral convexity view. **B** Midsagittal view. **C and D** Coronal views with the **C** section being more anterior to the **D** section. *ACC*: anterior cingulate cortex; *EBA*: extrastriate body area; *mPFC*: medial prefrontal cortex; *MST*: middle superior temporal area; *PCC*: posterior cingulate cortex; *PIVC*: parieto-insular vestibular cortex; *PMC*: premotor cortex; *pSTG*: posterior superior temporal gyrus; *SMA*: supplementary motor area; *TPJ*: temporoparietal junction; *VIP*: ventral intraparietal area; *vmPFC*: ventromedial prefrontal cortex. This figure was partially created in BioRender. Granno, S. (2025) [https://BioRender.com/br0aq7z].

Fig. 1 depicts and summarizes the different brain areas involved in self-related functions.

## 3 Perioperative and intraoperative assessment of self

### 3.1 Clinical assessment of self

The presence of a brain tumor can significantly affect high-level cognitive functions, including various dimensions of self-awareness. Under normal conditions, humans experience a sense of being a “real me”—the subject and agent of perception, action, and thought—a fundamental component of self-consciousness (Seth and Bayne, 2022). This experiential self is typically perceived as located within the physical boundaries of one's body, i.e. bodily self-consciousness (Ronchi et al., 2018). Tumor-related neural disruption, as well as surgical resection of infiltrated brain regions, can compromise several facets of self-awareness. In our clinical practice at the Geneva University Hospitals, we routinely assess these dimensions both pre- and postoperatively. This section outlines the most frequently evaluated and observed disturbances, including awareness of illness and bodily self-awareness disorders.

#### 3.1.1 Anosognosia

A primary and often underestimated aspect of compromised self-awareness is anosognosia—the partial or complete unawareness of one's own neurological or cognitive deficits. Originally described by Babinski in 1914 in patients with post-stroke hemiplegia, the term has since been expanded to encompass unawareness of various deficits resulting from acquired brain injuries (Babinski, 1914; Jenkinson and Fotopoulou, 2014).

In clinical settings, anosognosia is first evaluated through simple interviews, such as asking patients, “Why are you here?” or “What is the matter with you?“ (Cutting, 1978). For motor deficits, especially contralesional hemiplegia, awareness is probed with targeted questions about limb strength and direct feedback strategies (Bisiach et al., 1986). More structured tools include the Visual Analogue Test for Anosognosia for motor impairments (VATA-m), which uses pictorial tasks and a 4-point rating scale (Della Sala et al., 2009), as well as performance-based assessments like the Bimanual Task (Cocchini et al., 2010a), which evaluates whether patients integrate their motor deficits into their problem-solving strategies.

Cognitive anosognosia is typically inferred from the absence of spontaneous complaints despite evident deficits during neuropsychological tasks (Ronchi et al., 2014). Structured tools such as the Visual Analogue Test for Anosognosia for Language (VATA-L)(Cocchini et al., 2010b), the VATA for memory (VATA-mem) (Chapman et al., 2019), and the Prospective and Retrospective Memory Questionnaire (Crawford et al., 2006) are used to assess awareness of aphasia and memory impairments, respectively.

Another domain where anosognosia is frequently observed is unilateral spatial neglect. The most widely used assessment in clinical practice is the Catherine Bergego Scale, which evaluates discrepancies between patients' and caregivers’ ratings of spatial behaviors in daily activities (Azouvi, 1996). These awareness deficits are often modular—patients may be unaware of specific deficits while remaining aware of others (Jehkonen et al., 2000). In the context of tumor management, the presence of anosognosia has been well documented, with studies revealing a relatively high incidence. For instance, Lemaitre et al. (2021) reported anosognosia or anosodiaphoria in 17.35 % of patients in a prospective study of 98 individuals with low-grade glioma.

#### 3.1.2 Disorders of body awareness: personal neglect and disownership

Impairments of bodily self-awareness include personal neglect—reduced attention to the contralesional side of one's own body—and disownership—the experience that a limb or body part no longer belongs to oneself.

Personal neglect is assessed using tasks such as the Fluff Test (Cocchini and Jehkonen, 2001), the Vest Test (Glocker et al., 2006), or daily-life action tasks like combing hair (Pizzamiglio et al., 1992). These methods evaluate spontaneous, non-verbal bodily exploration, revealing neglect of the contralesional side even in the absence of verbal or visual cues.

Disownership, or somatoparaphrenia, is typically assessed via direct questioning (e.g., “Whose hand is this?“) (Bolognini et al., 2014), but newer tools also detect covert disownership via visual analogue scales (Ronchi et al., 2020). These scales have revealed subtle disturbances in body ownership even in patients who deny any deficit during interviews, highlighting the need for sensitive assessment methods (Cataldo et al., 2025)

#### 3.1.3 Neural correlates of self-awareness disorders

Neuroanatomically, anosognosia and bodily awareness disorders are not localized to a single region but arise from dysfunctions in distributed networks. Anosognosia for hemiplegia, for example, has been linked to damage in motor areas, the insula, PMC, thalamus, and parietal lobes, with diaschisis involving the hippocampus and memory systems contributing to the deficit (Berti et al., 2005; Vocat et al., 2010; Klingbeil et al., 2020).

Unawareness of aphasia and memory deficits has been associated with lesions in the left inferior frontal cortex and orbitofrontal regions, respectively (van der et al., 2021; Schnider, 2008). Anosognosia for spatial neglect typically involves the right inferior parietal and superior temporal regions (Vossel et al., 2013).

Personal neglect has been linked to damage in the right superior temporal gyrus, TPJ, insula, hippocampus, and thalamus, as well as disconnections involving the fornix and limbic system (Bertagnoli et al., 2022). Disownership, particularly in its overt forms, involves lesions in fronto-temporo-parietal areas and subcortical structures, including the insula, basal ganglia, and amygdala (Moro et al., 2023; Salvato et al., 2025). Covert disownership has recently been associated with bilateral disconnections in temporo-parietal and fronto-occipital networks (Ronchi et al., 2020; Cataldo et al., 2025).

Table 1 summarizes the main neural structures associated with the above-mentioned self-awareness disorders.

**Table 1:** Examples of self-awareness disorders and their neural correlates. *PMC*: premotor cortex; *TPJ*: temporoparietal junction.

Self-Awareness Disorder	Associated Lesioned Brain Areas
Anosognosia for hemiplegia	Motor areas, insula, PMC, thalamus, parietal lobes; diaschisis in hippocampus/memory systems
Anosognosia for aphasia	Left inferior frontal cortex
Anosognosia for memory deficits	Orbitofrontal cortex
Anosognosia for spatial neglect	Right inferior parietal lobule, right superior temporal gyrus
Personal neglect	Right superior temporal gyrus, TPJ, insula, hippocampus, thalamus; fornix and limbic disconnection
Overt disownership	Fronto-temporo-parietal cortex, insula, basal ganglia, amygdala
Covert disownership	Bilateral disconnections in temporo-parietal and fronto-occipital networks

Altogether, these findings underscore the modular and network-based nature of bodily self-awareness and its disorders—highlighting the importance of pre- and postoperative assessment tools for neurosurgical planning and outcome monitoring.

### 3.2 Neuroimaging of self: implications for neurosurgical planning

Advances in neuroimaging have allowed for increasingly precise mapping of brain networks, not only for classical functions like language or motor control but also for more complex and integrative processes such as self-related cognition. A landmark meta-analysis by Northoff et al. (2006) demonstrated that a distributed network of cortical midline structures (CMS) is consistently activated across self-referential tasks spanning verbal, emotional, spatial, and social domains. This supramodal self-network includes the mPFC, anterior and posterior cingulate cortices (ACC and PCC), precuneus, and retrosplenial cortex, each contributing to distinct yet interconnected aspects of self-processing—including self-evaluation, autobiographical memory, emotional awareness, and first-person perspective.

From a neurosurgical perspective, the consistent involvement of CMS across tasks suggests that lesions or resections involving medial prefrontal or cingulate regions may lead not only to cognitive deficits but also to alterations in personality, self-awareness, and interoceptive regulation—functions that are often underassessed in standard neurological exams but are crucial for post-surgical quality of life. In fact, these structures form the neuroanatomical core of what has been termed the “cortical-subcortical midline system”, integrating bodily and cognitive dimensions of the self through reciprocal projections to subcortical structures like the hypothalamus, periaqueductal gray, and brainstem nuclei.

Complementing these theoretical insights, recent evidence from a systematic review by Bonosi et al. (2023) underscores the importance of multimodal imaging for neurosurgical planning—especially when lesions are near functionally eloquent or self-related areas. Techniques such as fMRI, DTI with tractography, and intraoperative MRI (iMRI) allow for better delineation of tumor margins while preserving functionally and psychologically relevant brain regions.

For example, Luna et al. (2021) showed that preoperative fMRI mapping in tumor patients significantly reduced postoperative morbidity and enhanced cognitive outcomes. Henderson et al. (2020) emphasized that combining DTI-based tractography with fMRI enables better visualization of functional connectivity and plasticity, facilitating more assertive but safer resections. These findings support the idea that self-related networks—often embedded within the default mode network (DMN)—should be considered functionally eloquent and incorporated into standard preoperative assessment.

Moreover, these imaging methods can reveal individual differences in network organization and brain plasticity, allowing neurosurgeons to tailor interventions more precisely. This is particularly relevant in slowly progressing tumors like low-grade gliomas, where the brain has time to reorganize and shift self-related functions to adjacent areas, offering potential surgical windows of opportunity (Cargnelutti et al., 2020).

In summary, neuroimaging studies have identified a robust and distributed neural architecture for the self. These structures are now increasingly visible and quantifiable using advanced preoperative imaging. Neurosurgeons should be aware that resections involving CMS, particularly in the medial frontal and cingulate regions, carry risks of subtle but significant disruptions to self-consciousness. Incorporating functional imaging and connectivity analysis into surgical planning not only refines oncological precision but also contributes to a more holistic preservation of the human self.

### 3.3 Preserving white matter connectivity in self-referential networks

While most studies have focused on cortical hubs of self-processing, preserving the white matter connections between these areas is equally critical during surgery. Two sets of pathways–ventral stream pathways (Ng et al., 2021) and dorsal associative tracts (Herbet et al., 2014; Nakajima et al., 2018)–are considered high-priority targets for preservation. The ventral stream pathways–including the inferior fronto-occipital fasciculus (IFOF) and the inferior longitudinal fasciculus (ILF)–have been implicated to self-evaluative processing (Ng et al., 2021). Their disruption has been linked to impaired introspective abilities and semantic self-referential tasks (Ng et al., 2021). In parallel, dorsal associative tracts such as the cingulum, superior longitudinal fasciculus III (SLF III), fronto-striatal tract (FST), and the right arcuate fasciculus are essential for mentalizing–the capacity to represent one's own and others' mental states (Herbet et al., 2014; Nakajima et al., 2018). Damage to these tracts, especially in the right hemisphere, has been correlated with persistent high-level social cognition deficits.

While intraoperative brain shifts may alter the DTI position of these tracts and render neuronavigation obsolete, techniques routinely used in Geneva still allow their monitoring at all times during surgery. For instance, real-time neuronavigation updates are possible when using mixed reality injected into the operative microscope, by re-referencing the navigation on actual seen anatomical landmarks. Cortico-cortical evoked potentials (CCEPs) and subcortico-cortical evoked potentials (SCEPs) on these associative tracts also allow for their real-time assessment and integrity preservation, regardless of intraoperative brain shifts (for more detail, see Paragraph 3.3.2.).

### 3.4 Experimental assessment of the self: Self-Other Voice Discrimination (SOVD)

**Fig. 2:**
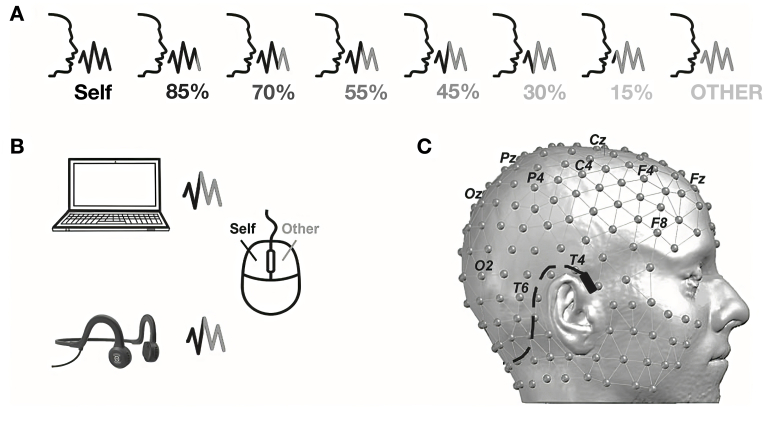
Self-Other Voice Discrimination (SOVD) task. **A** Different self-other voice morphs are presented multiple times to the participant (proportion of self-voice ranging from 15 % to 85 %). **B** The voice morphs are presented either using the laptop's speakers (air conduction) or a commercial headset (bone conduction) and the participants is instructed to respond whether the voice morph they hear resembles more their own voice or someone else's voice. **C** EEG setup with electrode names indicated in black. Adapted with permission from: Iannotti GR, Orepic P, Brunet D, Koenig T, Alcoba-Banqueri S, Garin DFA et al. EEG Spatiotemporal Patterns Underlying Self-other Voice Discrimination. *Cerebral Cortex*. 2021; 32 (9):1978–92. © Oxford University Press.

Self-voice processing offers a unique window into the perceptual and neural mechanisms underlying bodily and cognitive self-consciousness. The Self-Other Voice Discrimination (SOVD) paradigm (described in Fig. 2), developed in Geneva, combines voice morphing technology, bone and air conduction stimuli, and high-density EEG to investigate how individuals differentiate their own voice from that of others (Iannotti et al., 2021). In a cohort of healthy participants, the study identified a self-specific EEG microstate, emerging around 345 ms post-stimulus, which was more frequently activated during ambiguous voice trials dominated by self-voice content. This microstate was localized to a right-hemisphere dominant network encompassing the insula, cingulate cortex, hippocampus, amygdala, and temporal pole—regions closely associated with self-awareness, emotion, and interoception (Iannotti et al., 2021).

Crucially, the occurrence of this self-related map was inversely correlated with task accuracy and positively correlated with reaction times, suggesting that the more uncertain the brain is about whether the voice is “self,” the more this network is recruited. Furthermore, participants performed significantly better when hearing voice morphs through bone conduction, indicating the multisensory nature of self-voice perception—which likely involves not only auditory processing, but also somatosensory and vestibular signals (Iannotti et al., 2021).

Building on this work, a recent clinical study by Voruz et al. (2024) confirmed the discriminatory potential of the SOVD task in a cohort of neurosurgical patients with brain tumors or epileptogenic lesions. Using standardized clinical cut-offs derived from matched healthy controls, the study demonstrated that 17.65 % of patients exhibited selective impairments in self-voice recognition–suggestive of a specific self-related deficit not explainable by general cognitive or auditory dysfunction. Importantly, these impairments were not predicted by other neuropsychological measures or lesion lateralization, reinforcing the notion that self-voice discrimination constitutes a unique cognitive domain. In contrast, impaired other-voice discrimination was associated with deficits in inhibitory control, suggesting a distinct relationship between executive functions and non-self voice processing.

The SOVD task thus holds promise as a rapid (around 15 min), objective, and scalable neuropsychological tool for identifying self-related disturbances, which are otherwise difficult to quantify through standard clinical evaluation. Furthermore, the use of basic phonemic stimuli reduces semantic or linguistic confounds, making the task well suited for patients with language impairments. Given its neural specificity, behavioral sensitivity, and clinical feasibility, the SOVD task may serve as a valuable adjunct in preoperative mapping, intraoperative monitoring, and longitudinal neuropsychological assessment–especially in cases involving lesions near key self-processing hubs like the insula, mPFC, and cingulate cortex.

### 3.5 Self-related neural pathways and intraoperative electrophysiology of self

While neurosurgery has made substantial advances in preserving eloquent regions for movement, sensation, vision, and language, the self—a core dimension of human identity—remains largely unmapped in the operating room. Recent developments in neurophysiology, electrophysiology, and neuroimaging are beginning to define the neural circuits underlying self-consciousness, creating new opportunities to preserve and monitor the self intraoperatively (Schaller et al., 2021).

#### 3.5.1 Mapping the self: from networks to function

Functional imaging and intracranial studies have converged on a distributed network for self-processing that includes the mPFC, ACC, PCC, anterior insula, TPJ, inferior parietal lobule, and opercular cortex (Northoff et al., 2006; Blanke et al., 2015; Damasio et al., 2000). These regions support bodily self-consciousness through multisensory integration—including interoception—as well as higher-order cognitive self-functions such as self-reflection and self-representation (Craig, 2002; Blanke, 2012).

Lesions to these areas can disrupt personality, bodily self-awareness, theory of mind, or emotional regulation (Orepic et al., 2023; Campanella et al., 2014). Such disruptions highlight the importance of identifying and protecting these regions during surgery, particularly in tumors located near the frontal, temporal, and insular lobes (Schaller et al., 2021).

#### 3.5.2 Direct cortical and subcortical stimulation of self-related areas

Traditional intraoperative monitoring has focused on motor and sensory pathways, but techniques are evolving to allow for direct stimulation of self-related structures. In Geneva, new protocols have adapted subcortico-cortical evoked potentials (SCEPs) to map non-motor tracts, including visual pathways under general anesthesia (Boëx et al., 2021a; Schaller et al., 2021), but not self-related pathways yet.

Applying intraoperative neuromonitoring (IONM) methods traditionally used for motor and sensory functions also allows for the exploration of self-related pathways, particularly through intracranial electrical stimulation in areas such as the insula, TPJ, and mPFC. These sites have shown robust involvement in self-identification, interoception, and voice processing (Park et al., 2016; Babo-Rebelo et al., 2016; Mazzola et al., 2023). Intraoperatively, stimulation at or near these regions can produce measurable cardiac, emotional, or perceptual responses, reinforcing their dual roles in autonomic control and self-referential processing (Mazzola et al., 2023).

### 3.6 Heartbeat evoked potentials (HEPs)

A wide range of neurological functions can be assessed perioperatively using electrophysiological techniques. Motor evoked potentials (MEPs) and somatosensory evoked potentials (SSEPs) are routinely used in neurosurgical procedures involving eloquent motor and somatosensory areas, while direct cortical stimulation has expanded to include mapping of cranial nerves and cognitive functions such as language—even under general anesthesia (Martin et al., 2020). These tools are now indispensable for optimizing the onco-functional balance—maximizing tumor resection while preserving neurological function. However, no electrophysiological marker currently exists for mapping or monitoring the self, despite its fundamental importance for quality of life and post-surgical integrity. In recent years, Heartbeat-Evoked Potentials (HEPs)—also referred to as Heartbeat-Evoked Responses (HERs)—have emerged as promising biomarkers of bodily and cognitive self-consciousness, potentially offering a new physiological avenue for self-related monitoring in neurosurgery (Schaller et al., 2021).

HEPs are defined as cortical electrophysiological responses time-locked to the R-peak of the QRS complex in the electrocardiogram (ECG) and are typically recorded via electroencephalography (EEG), magnetoencephalography (MEG), or intracranial methods such as stereo-electroencephalography (SEEG). These signals reflect the integration of cardiac interoceptive inputs with ongoing brain activity, particularly in regions implicated in self-awareness, such as the anterior insula, ACC, and vmPFC(91, 94). HEPs have been associated with different levels of consciousness, showing decreased amplitude during unconscious states (such as deep sleep or general anesthesia) and increased amplitude during wakefulness or REM sleep—suggesting that HEPs may serve as neural indicators of conscious presence (Park and Blanke, 2019; Mazzola et al., 2023). It is worth noting that HEPs are modulated by the autonomic nervous system and are particularly sensitive to vagal afferent inputs and state-dependent arousal. For instance, studies have shown that HEP amplitude increases during heart rate deceleration, reflecting heightened parasympathetic (vagal) activity (Baranauskas et al., 2021).

Moreover, HEPs have been linked to conscious perceptual processing across sensory modalities. For example, higher HEP amplitudes have been shown to predict detection of near-threshold tactile, auditory, or visual stimuli, indicating a gating role in sensory awareness (Park et al., 2014). Crucially, HEP amplitude is modulated during experimental manipulations of bodily self-consciousness, such as the full-body illusion. When self-identification or self-location is experimentally altered—e.g., during visuo-tactile conflict in virtual reality—HEP amplitudes typically decrease, further confirming their association with bodily self-representation (Park et al., 2016; Babo-Rebelo et al., 2016).

Using SEEG, high-resolution HEP recordings have identified consistent sources in the insula, amygdala, temporal operculum, and frontotemporal cortex, regions central to interoception and the integration of self-related information. These findings suggest that HEPs may reflect both autonomic regulation and higher-order conscious processing, positioning them as ideal candidates for intraoperative monitoring of self-related networks (Mazzola et al., 2023).

Intracranial stimulation in epilepsy patients has already shown that HEPs can be modulated by stimuli related to self-identification (e.g., self-voice or body illusions), suggesting intraoperative feasibility (Schaller et al., 2021; Park and Blanke, 2019). Of note, in patients with refractory epilepsy, HEPs show significantly increased amplitudes and are strongly correlated with EEG power across multiple frequency bands, indicating altered heart-brain interaction and aberrant interoceptive processing (Melo et al., 2022).

## 4 Discussion

In clinical practice, the tools to assess self-awareness disturbances—such as anosognosia, personal neglect, and disownership—can still be improved. However, the ones described here, including the Fluff Test, VATA batteries, and new visual analogue scales for covert disownership (Seth and Bayne, 2022; Ronchi et al., 2014; Azouvi, 1996; Jehkonen et al., 2000), offer practical methods for integrating self-assessment into the standard neurosurgical pathway. These tests can serve as both diagnostic tools and outcome measures, paving the way for individualized, patient-centered neurosurgical care.

Neuropsychological changes after brain tumor resection often follow a non-linear course. For instance, early reductions in anxiety and depression may be followed by emerging memory impairments or cognitive complaints at 12 and 24 months, highlighting the importance of longitudinal follow-up (Dhandapani et al., 2017; Butterbrod et al., 2024; Armstrong et al., 1995). We recommend that self-related outcomes be assessed at baseline and again at 6, 12, and 24 months postoperatively, to account for both transient and delayed changes in cognition, emotional regulation, and self-perception.

In most neurosurgical practices, self-related neuropsychological deficits such as neglect syndrome, disownership, and anosognosia are very common in neuro-oncological patients. Even though only parts of the “self” are affected in these impairments, these patients experience significantly reduced quality of life and functional independence, with studies reporting strong negative correlations between neglect severity and domains such as mobility, self-care, and emotional well-being (Sobrinho et al., 2018; Gialanella et al., 2005; Franceschini et al., 2010). Furthermore, although the data on the topic is limited, professional reintegration remains a major challenge, with one review reporting that only 11 % of such patients successfully return to work (Bosma et al., 2020). Thus, monitoring and rehabilitating self-related neuropsychological impairments plays an important role in the patients’ quality of life.

In parallel to the electrophysiological approaches, the integration of advanced neuroimaging techniques into preoperative workflows offers another vital set of tools in preserving the self. fMRI and DTI now allow the visualization of core structures supporting bodily and cognitive self-processing—including the mPFC, anterior insula, TPJ, and PCC. These regions, embedded within the cortical midline system and the default mode network, are implicated in self-location, interoception, and affective processing (Northoff et al., 2006; Craig, 2002; Blanke, 2012). As shown in recent meta-analyses and tractographic studies, these areas are not only anatomically identifiable but also functionally eloquent and at risk during tumor resections (Klingbeil et al., 2020; van der et al., 2021; Schnider, 2008).

Moreover, the combination of imaging and IONM may allow for real-time risk stratification. Just as motor and language pathways are preserved through evoked potentials, emerging methods such as HEP-based monitoring, SOVD paradigms, and SCEPs now suggest the feasibility of protecting higher-order functions that underlie identity and agency (Schaller et al., 2021; Moro et al., 2023; Henderson et al., 2020). Given its robust neural specificity and behavioral sensitivity, the SOVD paradigm offers a promising tool for assessing self-related processing across the perioperative timeline. In combination with the application of electrophysiological markers like HEPs and direct evoked potentials to monitor self-related processes, these measurements can guide surgical planning. This is particularly true for tumors or epileptogenic zones located near self-processing hubs such as the insula, mPFC, or cingulate cortex (Iannotti et al., 2021). Furthermore, ongoing studies in Geneva have also proposed adapting standard tools such as the ultrasonic aspirator to deliver stimulation pulses while recording HEPs or evoked potentials in adjacent tissue (Boëx et al., 2021b).

These advances introduce a framework to preserve not only eloquent sensorimotor and cognitive domains, but the continuity of the patient's self—a key concern raised by both patients and clinicians. They challenge the traditional dichotomy between “eloquent” and “non-eloquent” cortex, arguing for a broader conceptualization of eloquence that includes the self.

Future work must refine the spatial specificity of HEP-based monitoring, determine its real-time applicability, and validate its clinical utility across patient populations. Looking forward, several research avenues are essential. First, there is a need for prospective, multicentric validation of HEP- and SOVD-based intraoperative monitoring protocols. Second, a functional risk map of self-related brain regions—similar to motor eloquence maps—should be developed and integrated into surgical planning software. Third, brain–heart interactions deserve more attention: as HEPs reflect both interoception and consciousness, monitoring them may inform not only surgical precision but also anesthetic depth and recovery.

## 5 Conclusion

The evolving field of surgical neuro-oncology increasingly recognizes that preserving the human self is as vital as preserving movement or speech. The work underway in Geneva demonstrates that the self can be mapped, monitored, and potentially protected using the tools of modern intraoperative neurophysiology. HEPs, self-related stimulation paradigms, and advanced neuroimaging integrated into perioperative care push the boundaries of what functional preservation means in neurosurgery.

Ultimately, within the concept of onco-functional balance, maximal safe resection is as crucial as the preservation of those neural circuits that sustain a patient's subjective sense of being. As the field continues to evolve, Geneva's interdisciplinary approach provides a compelling roadmap for neurosurgical teams worldwide—one that aligns surgical precision with the core of human experience.

## Declaration of generative AI and AI-assisted technologies in the writing process

During the preparation of this work, the authors used *ChatGPT (OpenAI)* for assistance with language refinement and synthesis of complex topics. After using this tool, the authors reviewed and edited the content as needed and take full responsibility for the content of the publication.

## Declaration of competing interest

The authors declare that they have no known competing financial interests or personal relationships that could have appeared to influence the work reported in this paper.
